# 2-Chloro-*N*-(3-chloro­phen­yl)benzamide

**DOI:** 10.1107/S1600536808018102

**Published:** 2008-06-19

**Authors:** B. Thimme Gowda, Sabine Foro, B. P. Sowmya, Hartmut Fuess

**Affiliations:** a Department of Chemistry, Mangalore University, Mangalagangotri 574 199, Mangalore, India; b Institute of Materials Science, Darmstadt University of Technology, Petersenstrasse 23, D-64287 Darmstadt, Germany

## Abstract

In the structure of the the title compound, C_13_H_9_Cl_2_NO, the N—H and C=O groups are mutually *trans*. Furthermore, the conformation of the C=O group is *syn* to the *ortho*-chloro group in the benzoyl ring, while the N—H bond is *anti* to the *meta*-chloro group in the aniline ring. The amide group forms dihedral angles of 89.11 (19) and 22.58 (37)°, respectively, with the benzoyl and aniline rings, while the benzoyl and aniline rings form a dihedral angle of 69.74 (14)°. The mol­ecules are linked into infinite chains through inter­molecular N—H⋯O hydrogen bonds.

## Related literature

For related literature, see: Gowda *et al.* (2003 ▶); Gowda, Foro *et al.* (2008 ▶); Gowda, Tokarčík *et al.* (2008 ▶).
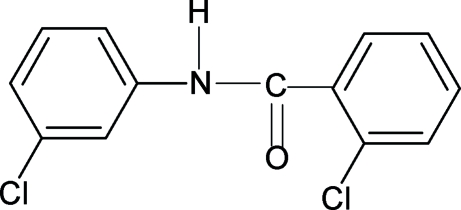


## Experimental

### 

#### Crystal data

C_13_H_9_Cl_2_NO
*M* *_r_* = 266.11
Orthorhombic, 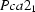

*a* = 11.430 (1) Å
*b* = 12.209 (2) Å
*c* = 8.878 (1) Å
*V* = 1238.9 (3) Å^3^
*Z* = 4
Mo *K*α radiation
μ = 0.51 mm^−1^
*T* = 299 (2) K
0.48 × 0.18 × 0.04 mm

#### Data collection

Oxford Diffraction Xcalibur diffractometer with a Sapphire CCD detector
Absorption correction: multi-scan (*CrysAlis RED*; Oxford Diffraction, 2007 ▶) *T*_min_ = 0.794, *T*_max_ = 0.980
4926 measured reflections
1746 independent reflections
1248 reflections with *I* > 2σ(*I*)
*R*_int_ = 0.022

#### Refinement

*R*[*F* ^2^ > 2σ(*F* ^2^)] = 0.038
*wR*(*F* ^2^) = 0.139
*S* = 1.15
1746 reflections
154 parameters
1 restraint
H-atom parameters constrained
Δρ_max_ = 0.39 e Å^−3^
Δρ_min_ = −0.42 e Å^−3^
Absolute structure: Flack (1983 ▶), 387 Friedel pairs
Flack parameter: 0.02 (13)

### 

Data collection: *CrysAlis CCD* (Oxford Diffraction, 2007 ▶); cell refinement: *CrysAlis RED* (Oxford Diffraction, 2007 ▶); data reduction: *CrysAlis RED*; program(s) used to solve structure: *SHELXS97* (Sheldrick, 2008 ▶); program(s) used to refine structure: *SHELXL97* (Sheldrick, 2008 ▶); molecular graphics: *PLATON* (Spek, 2003 ▶); software used to prepare material for publication: *SHELXL97*.

## Supplementary Material

Crystal structure: contains datablocks I, global. DOI: 10.1107/S1600536808018102/tk2274sup1.cif

Structure factors: contains datablocks I. DOI: 10.1107/S1600536808018102/tk2274Isup2.hkl

Additional supplementary materials:  crystallographic information; 3D view; checkCIF report

## Figures and Tables

**Table 1:** Hydrogen-bond geometry (Å, °)

*D*—H⋯*A*	*D*—H	H⋯*A*	*D*⋯*A*	*D*—H⋯*A*
N1—H1*N*⋯O1^i^	0.86	2.06	2.880 (5)	159

Symmetry code: (i) 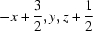.

**Fig. 1.**
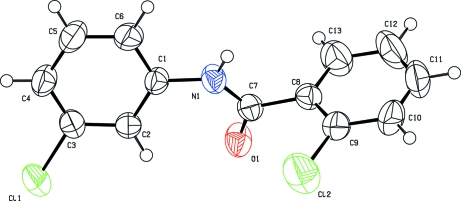
Molecular structure of (I), showing the atom labeling scheme. The displacement ellipsoids are drawn at the 50% probability level.

**Fig. 2.**
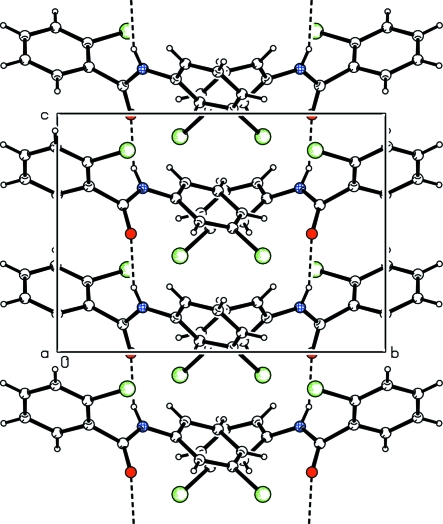
Molecular packing of (I) with hydrogen bonding shown as dashed lines.

## supplementary crystallographic information

### Comment

In the present work, the structure of 2-chloro-*N*-(3-chlorophenyl)-benzamide (I) has been determined to explore the effect of substituents on the structures of benzanilides (Gowda *et al.*, 2003; Gowda, Foro *et al.*, 2008; Gowda, Tokarčík *et al.*, 2008). The N—H and C=O bonds are *trans* to each other, Fig. 1, similar to that observed in *N*-(3-chlorophenyl)-benzamide (N3CPBA) (Gowda, Tokarčík *et al.*, 2008), 2-chloro-*N*-(phenyl)-benzamide (NP2CBA) (Gowda *et al.*, 2003), 2-methyl-*N*-(3-chlorophenyl)-benzamide (N3CP2MBA) (Gowda, Foro *et al.*, 2008), and other benzanilides. Further, the conformation of the C=O group is *syn* to the *ortho*-chloro group in the benzoyl ring, while the N—H bond is *anti* to the *meta*-chloro group in the aniline ring, similar to that observed in N3CP2MBA (Gowda, Foro *et al.*, 2008). The amide group forms dihedral angles of 89.11 (19)° and 22.58 (37)° with the benzoyl and aniline rings, respectively, while the benzoyl and aniline rings form a dihedral angle of 69.74 (14)°. These compare with the corresponding values of 55.8 (7)°, 18.6 (12)° and 37.5 (1)°, respectively, in N3CP2MBA. In the crystal structure of (I), the molecules are linked by N—H···O hydrogen bonds (Table 1) forming chains running along the *c* axis, Fig. 2.

### Experimental

Compound (I) was prepared according to the literature method (Gowda *et al.*, 2003). The purity of the compound was confirmed by melting point, and infrared and NMR spectra. Single crystals used for the X-ray diffraction analysis were obtained from an ethanolic solution of (I).

### Refinement

The H atoms were positioned with idealized geometries using a riding model with C—H = 0.93 Å, N—H = 0.86 Å, and with *U*_iso_(H) = 1.2*U*_eq_(C, N)

### Crystal data

**Table:**

C_13_H_9_Cl_2_NO	*F*_000_ = 544
*M**_r_* = 266.11	*D*_x_ = 1.427 Mg m^−^^3^
Orthorhombic, *P**c**a*2_1_	Mo *K*α radiation λ = 0.71073 Å
Hall symbol: P 2c -2ac	Cell parameters from 1634 reflections
*a* = 11.430 (1) Å	θ = 2.4–27.7º
*b* = 12.209 (2) Å	µ = 0.51 mm^−^^1^
*c* = 8.878 (1) Å	*T* = 299 (2) K
*V* = 1238.9 (3) Å^3^	Plate, colourless
*Z* = 4	0.48 × 0.18 × 0.04 mm

### Data collection

**Table:**

Oxford Diffraction Xcalibur diffractometer with a Sapphire CCD detector	1746 independent reflections
Radiation source: fine-focus sealed tube	1248 reflections with *I* > 2σ(*I*)
Monochromator: graphite	*R*_int_ = 0.022
*T* = 299(2) K	θ_max_ = 26.4º
Rotation method data acquisition using ω and φ scans	θ_min_ = 2.4º
Absorption correction: multi-scan(CrysAlis RED; Oxford Diffraction, 2007)	*h* = −14→7
*T*_min_ = 0.794, *T*_max_ = 0.980	*k* = −9→15
4926 measured reflections	*l* = −11→4

### Refinement

**Table:**

Refinement on *F*^2^	Hydrogen site location: inferred from neighbouring sites
Least-squares matrix: full	H-atom parameters constrained
*R*[*F*^2^ > 2σ(*F*^2^)] = 0.038	*w* = 1/[σ^2^(*F*_o_^2^) + (0.0797*P*)^2^ + 0.0826*P*] where *P* = (*F*_o_^2^ + 2*F*_c_^2^)/3
*wR*(*F*^2^) = 0.139	(Δ/σ)_max_ = 0.002
*S* = 1.15	Δρ_max_ = 0.39 e Å^−^^3^
1746 reflections	Δρ_min_ = −0.42 e Å^−^^3^
154 parameters	Extinction correction: none
1 restraint	Absolute structure: Flack (1983), 387 Friedel pairs
Primary atom site location: structure-invariant direct methods	Flack parameter: 0.02 (13)
Secondary atom site location: difference Fourier map	

### Special details

**Table:**

Geometry. All e.s.d.'s (except the e.s.d. in the dihedral angle between two l.s. planes) are estimated using the full covariance matrix. The cell e.s.d.'s are taken into account individually in the estimation of e.s.d.'s in distances, angles and torsion angles; correlations between e.s.d.'s in cell parameters are only used when they are defined by crystal symmetry. An approximate (isotropic) treatment of cell e.s.d.'s is used for estimating e.s.d.'s involving l.s. planes.
Refinement. Refinement of *F*^2^ against ALL reflections. The weighted *R*-factor *wR* and goodness of fit *S* are based on *F*^2^, conventional *R*-factors *R* are based on *F*, with *F* set to zero for negative *F*^2^. The threshold expression of *F*^2^ > σ(*F*^2^) is used only for calculating *R*-factors(gt) *etc*. and is not relevant to the choice of reflections for refinement. *R*-factors based on *F*^2^ are statistically about twice as large as those based on *F*, and *R*- factors based on ALL data will be even larger.

### Fractional atomic coordinates and isotropic or equivalent isotropic displacement parameters (Å^2^)

**Table:**

	*x*	*y*	*z*	*U*_iso_*/*U*_eq_	
Cl1	0.75422 (12)	0.62626 (9)	−0.0970 (2)	0.0776 (5)	
Cl2	0.49999 (15)	0.21361 (13)	0.3455 (3)	0.1038 (7)	
O1	0.6539 (3)	0.2242 (3)	−0.0041 (3)	0.0597 (8)	
N1	0.7788 (3)	0.2603 (3)	0.1873 (4)	0.0499 (9)	
H1N	0.8049	0.2334	0.2702	0.060*	
C1	0.8275 (3)	0.3614 (3)	0.1443 (5)	0.0437 (9)	
C2	0.7719 (3)	0.4339 (3)	0.0469 (5)	0.0433 (9)	
H2	0.7012	0.4159	0.0014	0.052*	
C3	0.8252 (4)	0.5339 (3)	0.0200 (5)	0.0481 (10)	
C4	0.9300 (4)	0.5626 (4)	0.0814 (5)	0.0559 (12)	
H4	0.9638	0.6301	0.0602	0.067*	
C5	0.9845 (4)	0.4894 (4)	0.1753 (7)	0.0680 (14)	
H5	1.0566	0.5072	0.2173	0.082*	
C6	0.9340 (4)	0.3903 (4)	0.2082 (6)	0.0577 (11)	
H6	0.9713	0.3422	0.2738	0.069*	
C7	0.6971 (4)	0.2001 (3)	0.1166 (5)	0.0444 (9)	
C8	0.6619 (3)	0.0962 (3)	0.1951 (5)	0.0458 (9)	
C9	0.5699 (4)	0.0925 (4)	0.2959 (6)	0.0578 (12)	
C10	0.5336 (4)	−0.0053 (4)	0.3608 (7)	0.0766 (16)	
H10	0.4709	−0.0067	0.4275	0.092*	
C11	0.5915 (6)	−0.0989 (4)	0.3252 (9)	0.0805 (17)	
H11	0.5689	−0.1646	0.3696	0.097*	
C12	0.6820 (6)	−0.0983 (4)	0.2255 (9)	0.090 (2)	
H12	0.7198	−0.1633	0.2007	0.108*	
C13	0.7178 (5)	−0.0001 (4)	0.1608 (8)	0.0771 (15)	
H13	0.7803	0.0003	0.0937	0.093*	

### Atomic displacement parameters (Å^2^)

**Table:**

	*U*^11^	*U*^22^	*U*^33^	*U*^12^	*U*^13^	*U*^23^
Cl1	0.0812 (7)	0.0536 (6)	0.0981 (10)	−0.0008 (6)	−0.0181 (7)	0.0268 (7)
Cl2	0.1036 (10)	0.0798 (9)	0.1282 (16)	0.0244 (8)	0.0548 (10)	0.0169 (10)
O1	0.0753 (19)	0.0604 (18)	0.0433 (16)	−0.0176 (15)	−0.0119 (16)	0.0148 (15)
N1	0.059 (2)	0.0434 (17)	0.047 (2)	−0.0080 (15)	−0.0111 (17)	0.0101 (17)
C1	0.043 (2)	0.046 (2)	0.042 (2)	−0.0078 (16)	0.0003 (19)	0.007 (2)
C2	0.044 (2)	0.044 (2)	0.042 (2)	−0.0025 (17)	−0.0062 (19)	0.0029 (18)
C3	0.054 (2)	0.040 (2)	0.051 (3)	0.0010 (18)	0.004 (2)	0.0030 (19)
C4	0.053 (3)	0.060 (3)	0.055 (3)	−0.017 (2)	−0.001 (2)	0.011 (2)
C5	0.051 (2)	0.078 (3)	0.075 (4)	−0.023 (2)	−0.008 (3)	0.018 (3)
C6	0.050 (2)	0.068 (3)	0.056 (3)	−0.005 (2)	−0.011 (2)	0.018 (2)
C7	0.047 (2)	0.046 (2)	0.040 (2)	−0.0003 (18)	0.0013 (19)	0.0077 (19)
C8	0.047 (2)	0.045 (2)	0.046 (2)	−0.0003 (16)	−0.007 (2)	0.0032 (18)
C9	0.050 (2)	0.059 (3)	0.064 (3)	−0.002 (2)	0.001 (2)	0.014 (2)
C10	0.063 (3)	0.078 (3)	0.089 (4)	−0.022 (3)	0.008 (3)	0.029 (4)
C11	0.093 (4)	0.053 (3)	0.096 (4)	−0.020 (3)	−0.016 (4)	0.027 (3)
C12	0.113 (5)	0.042 (3)	0.116 (5)	0.013 (3)	0.003 (5)	0.011 (3)
C13	0.085 (3)	0.056 (3)	0.090 (4)	0.009 (2)	0.013 (4)	0.002 (3)

### Geometric parameters (Å, °)

**Table:**

Cl1—C3	1.735 (4)	C5—H5	0.9300
Cl2—C9	1.737 (5)	C6—H6	0.9300
O1—C7	1.217 (5)	C7—C8	1.502 (6)
N1—C7	1.344 (5)	C8—C13	1.372 (6)
N1—C1	1.406 (5)	C8—C9	1.383 (6)
N1—H1N	0.8600	C9—C10	1.389 (7)
C1—C6	1.389 (6)	C10—C11	1.358 (8)
C1—C2	1.391 (6)	C10—H10	0.9300
C2—C3	1.385 (5)	C11—C12	1.362 (9)
C2—H2	0.9300	C11—H11	0.9300
C3—C4	1.362 (6)	C12—C13	1.390 (8)
C4—C5	1.372 (7)	C12—H12	0.9300
C4—H4	0.9300	C13—H13	0.9300
C5—C6	1.372 (6)		
			
C7—N1—C1	128.9 (3)	O1—C7—N1	124.1 (4)
C7—N1—H1N	115.5	O1—C7—C8	120.3 (4)
C1—N1—H1N	115.5	N1—C7—C8	115.6 (3)
C6—C1—C2	119.5 (3)	C13—C8—C9	118.0 (4)
C6—C1—N1	117.3 (3)	C13—C8—C7	119.8 (4)
C2—C1—N1	123.1 (3)	C9—C8—C7	122.1 (4)
C3—C2—C1	117.9 (4)	C8—C9—C10	121.6 (5)
C3—C2—H2	121.1	C8—C9—Cl2	119.1 (3)
C1—C2—H2	121.1	C10—C9—Cl2	119.3 (4)
C4—C3—C2	123.0 (4)	C11—C10—C9	118.8 (5)
C4—C3—Cl1	118.9 (3)	C11—C10—H10	120.6
C2—C3—Cl1	118.1 (3)	C9—C10—H10	120.6
C3—C4—C5	118.4 (4)	C10—C11—C12	121.1 (5)
C3—C4—H4	120.8	C10—C11—H11	119.4
C5—C4—H4	120.8	C12—C11—H11	119.4
C4—C5—C6	120.8 (4)	C11—C12—C13	119.8 (5)
C4—C5—H5	119.6	C11—C12—H12	120.1
C6—C5—H5	119.6	C13—C12—H12	120.1
C5—C6—C1	120.4 (4)	C8—C13—C12	120.7 (5)
C5—C6—H6	119.8	C8—C13—H13	119.7
C1—C6—H6	119.8	C12—C13—H13	119.7
			
C7—N1—C1—C6	−160.3 (4)	N1—C7—C8—C13	−92.5 (5)
C7—N1—C1—C2	22.4 (7)	O1—C7—C8—C9	−90.1 (5)
C6—C1—C2—C3	−1.1 (6)	N1—C7—C8—C9	91.1 (5)
N1—C1—C2—C3	176.2 (4)	C13—C8—C9—C10	−0.4 (7)
C1—C2—C3—C4	1.6 (6)	C7—C8—C9—C10	176.0 (5)
C1—C2—C3—Cl1	−178.2 (3)	C13—C8—C9—Cl2	178.2 (4)
C2—C3—C4—C5	−0.7 (7)	C7—C8—C9—Cl2	−5.3 (6)
Cl1—C3—C4—C5	179.2 (4)	C8—C9—C10—C11	0.9 (8)
C3—C4—C5—C6	−0.8 (8)	Cl2—C9—C10—C11	−177.8 (5)
C4—C5—C6—C1	1.3 (8)	C9—C10—C11—C12	−1.3 (9)
C2—C1—C6—C5	−0.3 (7)	C10—C11—C12—C13	1.2 (10)
N1—C1—C6—C5	−177.7 (5)	C9—C8—C13—C12	0.3 (9)
C1—N1—C7—O1	2.6 (7)	C7—C8—C13—C12	−176.2 (5)
C1—N1—C7—C8	−178.6 (4)	C11—C12—C13—C8	−0.8 (10)
O1—C7—C8—C13	86.2 (6)		

### Hydrogen-bond geometry (Å, °)

**Table:**

*D*—H···*A*	*D*—H	H···*A*	*D*···*A*	*D*—H···*A*
N1—H1N···O1^i^	0.86	2.06	2.880 (5)	159

Symmetry codes: (i) −*x*+3/2, *y*, *z*+1/2.
